# Codeveloping a Set of Quality of Care Indicators for People Living With Multiple Long-Term Conditions in Primary Care: Protocol for a Modified RAND/UCLA Study

**DOI:** 10.2196/99217

**Published:** 2026-08-07

**Authors:** Sara Tavares, Thomas Beaney, Arad Reisberg, Ania Henley, Angela Edwards, David Belsey, Laura Downey

**Affiliations:** 1 The George Institute for Global Health, Imperial College London, Scale Space, 58 Wood Ln, London, England, W12 7RZ, United Kingdom, 44 07568150992; 2 Patient Experience Research Centre (PERC), Imperial College London, London, United Kingdom; 3 The George Institute for Global Health, Imperial College London, Sydney, Australia; 4 Health Translation Hub, The George Institute for Global Health, UNSW Sydney, Sydney, New South Wales, Australia

**Keywords:** quality indicators, primary care, multiple long-term conditions, multimorbidity, patient and public involvement, co-design, RAND/UCLA appropriateness method

## Abstract

**Background:**

Quality indicators in primary care remain predominantly disease-specific and professionally defined, with limited incorporation of what matters most to people living with multiple long-term conditions (MLTCs) and their caregivers. Existing frameworks and quality standards provide important conceptual direction, but few produce a pragmatic set of ready-to-use indicators.

**Objective:**

This study aims to codevelop a disease-agnostic set of quality of care indicators with people living with MLTCs, caregivers, and health care professionals, including preliminary operational specifications to support future feasibility testing and service improvement in general practices within the National Health Service in England.

**Methods:**

This protocol describes a 3-round modified RAND/UCLA Appropriateness Method (RAM) study. The first round will evaluate the importance of candidate indicators through an online questionnaire to an expert panel of patients, caregivers, and health care professionals. Indicators without agreement or requiring revision will proceed to a structured consensus meeting (round 2) with subsequent rerating rounds (round 3) for feasibility and measurability.

**Results:**

As of July 2026, 10 patient and caregiver participants and 12 health care professionals have consented to join an expert panel. The expected RAM study completion, including data analysis and reporting, is early 2027. Fifty-four candidate quality indicators have been generated through triangulation of findings between a scoping review of published quality indicators in MLTCs and a qualitative study with 21 patients and caregivers with lived experience of MLTCs. Ongoing patient and public involvement and engagement with four community partners have further informed the study design, interpretation of multistage findings, and refinement of candidate indicators. The indicators have been mapped to a structure-process-outcome quality framework and will be taken forward into a modified 3-staged RAM study.

**Conclusions:**

This study will generate a co-designed and implementation-oriented set of primary care quality indicators for MLTCs. The final indicator set is intended to support future measurement, quality improvement, and later field testing in real-world primary care systems.

## Introduction

The coexistence of 2 or more long-term (chronic) conditions, commonly referred to as multiple long-term conditions (MLTCs) or multimorbidity, is a major global public-health problem with rates expected to rise over the next 30 years as populations live longer [1]. MLTCs affect populations across low, middle, and high-income countries [2], with global estimates suggesting that around 37.2% of adults living in the community are affected [3]. The prevalence of MLTCs increases with age, although the greatest number of people affected are those under 65 years old [3]. People with MLTCs are more likely to experience difficulties accessing and navigating health care services [4], functional limitations, poorer quality of life, reduced ability to work, and lower life expectancy [5]. MLTCs increase demand on health systems, particularly within primary care, where most receive their care [6]. People living with MLTCs also encounter unique challenges such as fragmented care with limited continuity between multiple health care providers, as well as poor coordination across services [7]. Subanalysis of patient experience surveys in primary care suggests that satisfaction with care declines as the number of conditions increases, and perceptions of care quality may differ from those without MLTCs [8].

To identify areas where the quality of care can be improved, collecting and interpreting health care performance data are crucial. Quality indicators, often referred to as quality measures or metrics, are one way to achieve this, by supporting the monitoring of the components of care associated with quality [9]. Rich evidence is available on the treatment burden for people with MLTCs [10,11], interventions to improve care [12,13], or individual components of care for chronic conditions [14,15]. However, limited consensus persists on which quality indicators best capture the quality of primary care delivered to people living with MLTCs [14]. Internationally published indicator sets for care quality in primary care often lack operational detail on definitions, numerators, denominators, and data sources, limiting direct transferability to current clinical care settings [16].

In the United Kingdom, while the National Institute for Health and Care Excellence (NICE) has produced a set of quality indicators intended to capture the processes of care relevant to those living with MLTCs, these have not been implemented through the national pay-for-performance program implemented in general practices (GPs) [17]. Through this program, payments to GPs are made according to the achievement of disease-specific quality indicator targets [18]. As a result, quality of care for people living with MLTCs continues to be monitored through aggregated analysis of these disease-specific Quality Outcomes Framework (QOF) indicators [18]. The limitation of these single-disease indicators is that they do not adequately reflect the complexity of living or caring for people with MLTCs, whose care and lived experience is not only shaped by their chronic conditions, but by an interplay of social, economic, and physical variables [19,20].

For care provider institutions, it is particularly challenging to measure holistic care that is relevant to patients and caregivers [21,22]. This is because a quality indicator needs to be able to measure a precisely defined activity for it to be reliable. Unlike clinical outcomes of care (eg, hospitalization or mortality), processes of care (eg, interpersonal relationships) are not consistently codified into standardized data fields within electronic health records and are often inserted within unstructured narratives, limiting their routine data extraction [23,24].

We aim to codevelop a set of quality indicators that better reflect the complexities of MLTC care, moving beyond the use of aggregated disease-specific metrics as measures of quality. Combining existing evidence, lived experience, and indicator development methodology via structured consensus, this study aims to codevelop a disease-agnostic set of quality-of-care indicators with people living with MLTCs, caregivers, and health care professionals, including definitions, preliminary numerators, denominators, exclusions, and data sources to support future feasibility testing and service improvement in primary care settings within the National Health Service in England.

## Methods

### Study Design

This study will adopt a modified RAND/UCLA Appropriateness Method (RAM) Delphi design, a structured consensus method widely used to develop and validate quality indicators across health care settings, including in psycho-oncological care [25], primary care for older adults [26], specialist mental health referral pathways [27], delivery of person-centered care [28], and various single-disease indicators [29-31]. By combining evidence from the literature with structured expert judgment, RAM supports the development of indicators that are evidence-informed, transparent, and reproducible, while strengthening face and content validity through consensus. In addition, its flexible format allows the feasibility of data collection questions, which is crucial to guide future clinical implementation strategies [9,32,33].

This RAM study will comprise multiple components: (1) a web-based first-round rating of candidate indicators; (2) a structured hybrid consensus meeting to discuss indicators not reaching agreement during round 1; and (3) a final rerating round to obtain final consensus on definitions, importance, and feasibility of data collection using routine primary care electronic records. When completed, the final report of this RAM study will follow the Delphi recommendations for an interdisciplinary standardized reporting (DELPHISTAR) [34].

### Patient and Public Involvement

This study is underpinned by a pragmatic-interpretivist paradigm, which recognizes that knowledge intended to improve health care must be both grounded in multiple perspectives and lived experience while focusing on practical and applicable solutions for real-world practice [35]. Pragmatism supports the use of multiple methods, data sources, and forms of expertise to address the research question and support future implementation [36,37], while interpretivism emphasizes understanding how the subjective experiences of patients and caregivers shape knowledge and perceptions of care quality in the context of everyday life [38].

This study will be explicitly grounded in principles of coproduction, collaboration, and inclusion, adopting an approach to conducting research *with* patients and caregivers rather than *for* them [39]. Their active participation during this RAM study will ensure that emerging indicators remain meaningful to those affected by MLTCs and relevant to clinical practice. Previous and future contributions from our community partners (CPs) are reported in detail following the Guidance for Reporting Involvement of Patients and the Public (GRIPP) framework [40] (Checklist 1) ().

### Conceptual Framework Through a Scoping Review and Qualitative Interviews

The first step in indicator development requires a conceptual framework and rigorous methods to identify, clarify, and define what concepts and dimensions of quality should be measured [32,41,42]. An international scoping review is published elsewhere [43], and it synthesized evidence on the processes and content of existing MLTC-related quality indicators, including the extent to which patients had been involved in their development. Seventy-eight disease-agnostic indicators were extracted eliciting patient and/or caregiver priorities, and 33 quality domains were synthesized for future indicator development. Across studies, quality was more commonly articulated through processes of care than through organizational structures or outcomes, with recurring emphasis on coordination, continuity, shared decision-making, and holistic and tailored assessments. Few indicators were fully operationalized for routine primary care use, and many were measured through patient-experience surveys rather than clearly specified indicators with defined data sources or measurement rules.

Second, interviews with 21 adults living with or caring for someone with MLTCs explored how high-quality care is understood from lived experience. Through reflexive inductive thematic analysis, four interrelated domains of quality emerged: optimal clinical care, patient and carer centeredness, organizational processes and access, and system preparedness and support. This qualitative work showed that health care quality was dynamic, with priorities shifting over time according to symptoms, treatment burden, care transitions, and changing life circumstances. The full manuscript is under review elsewhere.

### Panel Eligibility and Selection

Recommendations from the RAM suggest a panel size of between 7 and 15 members to allow a focused discussion, with the opportunity for different perspectives to be expressed [33]. We will aim to recruit approximately 15 panel members, with a planned stakeholder composition of at least 7 patients and/or caregivers, 3 health care professionals from different professional roles, 1 commissioner, 1 service or operational manager, and 2 researchers or clinical academics. This planned composition is intended to ensure that patients, caregivers, and community advisory partners together represent at least half of the panel. Although there is no universal standard definition for who qualifies as an expert panel member in Delphi studies [44], we will consider the following as expert panelists: health care professionals (HCPs), commissioners, managers, or researchers with at least 5 years of experience relevant to primary care provision, quality improvement, or MLTC care. Publication history will not be an eligibility criterion because this study considers implementation and service insight alongside methodological or theoretical knowledge [45]. As such, we will include medical professionals, allied professionals, and nurses, if experience was established through clinical practice, organizational leadership, policy work, or academic expertise. The mixed sample of panelists background and expertise will account for a wide variation of relevant views to the care of those with MLTCs, avoiding homogeneous approaches to quality of care in MLTCs and mitigating cognitive biases [45].

All 21 patients and caregivers who participated in the earlier qualitative interviews will be invited to take part in the RAM panel, supporting the continuity of involvement across this multistep study, and to help reduce attrition between rounds. These participants have lived experience of MLTCs and receive care across primary and secondary care services. Many are also engaged in local community activities, patient-led charities, or research involvement initiatives. If necessary, the recruitment of additional patient and caregiver participants will be supplemented through the networks of our community advisory partner and Imperial’s Patient Experience Research Centre, including engagement with local community organizations and patient-led voluntary groups. Patients and caregivers will be eligible if they are aged 18 years or older, have relevant self-reported lived experience of living with or caring for someone with MLTC, are able to provide informed consent and participate in the rating rounds and consensus meetings. Commissioners and service managers will be identified through repeated engagement with the local Integrated Care Board network. Health care professionals and clinical academics will be approached via purposive and snowballing approaches from the research team’s academic and professional networks.

All participants will be compensated for their time with a high-street voucher at the completion of each round. To reduce attrition, we will provide clear information on expected time commitment for each round, schedule round 2 consensus meeting with at least 4 weeks in advance, send weekly reminders for the completion of surveys, and offer technical support. Between all rounds, HCP participants will receive data synthesis reports, while patient and caregiver participants will receive lay summaries of the findings.

Although we aim to retain a consistent core panel across this study’s rounds to ensure that participants can consider group feedback and rerate the same items over time [46], we will also allow new panel members who were unable to participate in round 1 to join the consensus meeting in round 2. Panelists who missed a previous round will continue to be invited to participate unless they have expressly withdrawn from the study. Boel et al [47] found that retaining these panelists does not significantly affect Delphi outcomes and may improve response rates. If additional or replacement panelists are recruited between rounds, recruitment will be purposive and monitored to preserve the balance of the panel. Where possible, participants will be recruited from the same stakeholder group (ie, pharmacists, nurses, or GPs) and will have broadly comparable professional background or lived experience of MLTCs to those who have withdrawn. Changes in panel composition across rounds will be reported transparently in the study flow diagram and participant characteristics table.

### Preparing for Round 1: Web-Based Survey

The findings from both the scoping review and the qualitative interviews were triangulated and mapped onto a structure-process-outcome framework informed by the Donabedian model (Table S1 in Multimedia Appendix 1) [48]. This identified areas of convergence between published evidence and qualitative lived experience, identifying the main domains of care quality and the existing indicators developed in the literature.

To develop indicators, the RAM does not prescribe a specific syntactic format and suggests that items are expressed as clinical scenarios or system attributes that can be judged for rating [33]. At this stage, 54 candidate indicators were drafted as clear declarative statements. Indicators following traditional “If-Then” definitions [9] were intentionally avoided to support a balanced assessment of validity, importance, and redundancy without eliciting premature operational framing.

Traditional RAM panels often use appropriateness as the main rating construct, defined by whether the expected health benefits of an intervention outweigh its potential harms [26,33]. Because our indicators relate to structures and processes of primary care, rather than discrete single-disease interventions, during this round 1 all participants will be asked to vote on the importance of candidate indicators, rather than on appropriateness. Importance will be defined as the extent to which an indicator addresses an aspect of care encountered in routine primary care and is linked to care components that matter to patients, caregivers, quality improvement, and primary care clinical activities [32,42,49].

Guidance on rating quality indicators is extremely heterogeneous without a universally used set of questions for its assessment, but there is agreement that robust indicator development should at minimum address content and face validity (Table 1) [9,32,42,50]. Validity can be assessed using varying methods, and it usually asks patients or HCPs about the relevance, comprehensiveness, and comprehensibility of the items [33,49].

**Table 1.** Criteria for indicator rating and assessment.

Criteria for indicator rating and assessment	Description
Content validity	The extent to which the indicator accurately measures what it is intended to measure is underpinned by evidence and captures meaningful aspects of the quality of care, consistent with professional knowledge and the outcomes of high-quality care [32,42,49,50].
Face validity	The extent to which the indicator makes sense logically and clinically [50], often reflected as to whether they link with structure, processes, and/or outcomes in the health care system [32]. Campbell et al [9] defines it as whether any indicators were rated by panels and if there is evidence that the indicator is underpinned by consensus.
Relevance, importance, or acceptability	These terms are usually used interchangeably in published studies developing indicators. Relevance can describe either the impact of disease on health expenditure and organizational contexts. Also relates to the stakeholders’ views on its importance as a quality gap or potential for performance improvement as a gap in performance [27,51]. Acceptability describes the extent to which indicator contents are acceptable to those being assessed and those undertaking the assessment. It is primarily used during application of quality indicators, rather than development stages [9].
Redundancy	Although many methodological recommendations focus on single indicators, indicator sets require explicit attention to omission, redundancy, overrepresentation of some domains, inclusion of irrelevant indicators [42], or existing related and competing measures already in practice [52].

Patients and caregivers will rate the importance based on their lived experience of MLTCs, while HCPs will additionally rate the importance of indicators for measuring overall quality of care and to primary care clinical activities. Ordinal ratings will be used following a 9-point Likert scale, aligning with RAM recommendations facilitating granular measurement [33,53].

Advised by our CPs, patients and caregivers will not be asked to vote importance to quality improvement or primary care clinical activities, as these constructs were considered less accessible and relevant from lived experience alone. Table 2 summarizes round 1 rating assessment criteria that will be asked to each participant group.

**Table 2.** Round 1 rating domains by participant group for each indicator.

Participant group and rating assessment criteria	Questions	Rating scale
Patients and caregivers		
Importance to patients and caregivers (face validity)	How important is this indicator to patients and caregivers affected by MLTCs^a^?	Nine-point Likert scale for importance, where 1 indicates “extremely unimportant” and 9 indicates “extremely important”
Comprehensiveness or clarity	Is the definition of this indicator clear enough to understand?	Yes, no, or free text
Redundancy	To what extent does this indicator reflect current routine clinical practice?	Nine-point Likert scale, where 1 indicates “does not reflect routine practice,” 5 indicates “partially reflects routine practice,” and 9 indicates “very accurately reflects routine practice”
Health care professionals		
Importance	How important is this indicator to patients and caregivers affected by MLTC?	Nine-point Likert scale for importance
Content validity	How important is this indicator to measure high quality of care related to its targeted domain of care (eg, respectful care)?	Nine-point Likert scale for importance
Face validity	How important is this indicator to primary care clinical activities or practice?	Nine-point Likert scale for importance
Redundancy	To what extent does this indicator reflect current routine clinical practice? Are there existing indicators measuring the same domain of care quality currently in use in your practice?	Yes, no, or free text Nine-point Likert scale for routine practice
Comprehensiveness or clarity	Is the definition of this indicator clear enough to understand?	Yes, no, or free text

a MLTC: multiple long-term condition.

To minimize redundancy and avoid future indicator burden [42,52], HCPs will also be asked on whether an indicator is already covered by existing metrics, indicators, or digital systems in current practice. To complement this question, we will inquire whether the indicator quality domain is already being implemented in local clinical practice.

Both patients and HCPs will be provided with a question to comment on the clarity of indicator definition. This will be supplemented by the opportunity to provide written comments and to suggest additional indicators based on their expertise [54]. Clarity will be given as a quality check and not a consensus construct; therefore, it will be voted on in a dichotomous manner (yes, no, or needs changing) instead of a Likert-type scale.

Round 1 will be conducted remotely using an online survey platform Qualtrics, following recommendations for web-based RAM panels [33]. Before rating the indicators, participants will receive information on the previous scoping review, qualitative interview findings, and specific instructions on how to rate the indicators in the first pages of the Qualtrics survey [33]. This ensures that all panel members are familiar with the rating and ranking process, as well as common definitions used throughout the survey. At this stage, panelists will be specifically instructed not to consider feasibility or implementation barriers, such as workload constraints, cost implications, or existing local service configurations, when making their ratings.

All participants will be given up to 5 weeks to respond, with automated reminders sent after 2 weeks of inactivity.

### Between Rounds 1 and 2: Data Analysis

Consensus thresholds will be informed by RAM, and ratings will be summarized using medians and IQRs for each indicator and each question. Medians and IQRs will be calculated separately for each stakeholder group given the heterogeneous nature of the panel combining lived-experience and professional perspectives.

Disagreement will be defined as evidence of polarization, whereby at least one-third of the total panel rates an indicator in the 1‐3 region and at least one-third rates the same indicator in the 7‐9 region [33].

The IQR will be used as a measure of dispersion to assess the degree of agreement among panel members and will not be used as a strict threshold for consensus [34]. Lower IQR values (eg, IQR ≤2) suggest greater consensus, and high IQR values (eg, IQR ≥4) indicate wider dispersion. In the absence of disagreement, final indicator decisions will be based on median scores as follows [33,34]:

- Indicator will be *accepted* and proceed straight to round 3: if both stakeholder-group median ratings are between 7 and 9 across rating assessment criteria questions, with no disagreement.
- Indicator will be classified as *“uncertain” and retained for round 2 discussion*: if any stakeholder-group median is 4 to 6, if disagreement is present, or if stakeholder groups are discordant, for example, if one group rates the indicator on a median between 7 to 9 and the other group rates it below a median of 6 in any question.
- Indicator will be *rejected and excluded* from subsequent rounds: if both stakeholder-group medians rating are between 1 and 3, with no disagreement.

Written comments from free-text questions will be analyzed through content analysis methods to identify refinement needed in the definitions provided or overlap with existing systems indicating redundancy [45]. In line with recommendations for assessing the quality of Delphi studies, the volume and depth of qualitative responses (eg, presence of substantive, explanatory comments) will be examined as an indicator of participant engagement [45,55]. Based on these comments, indicators may be merged, reworded, or excluded. Indicators will be retained for round 2 discussion if free-text comments indicate the need for discussion, conceptual overlap between indicators, or limited added value to lived experiences or clinical practice, even if they are rated as highly important by any stakeholder group. Sensitivity analyses will be conducted to assess the robustness of the findings, including comparisons of indicator ratings between patients and HCPs. Where relevant, to assess potential nonresponse bias, a post hoc Mann-Whitney *U* test comparing early and late responders, using median indicator ratings, will be conducted [56].

To address context-specific operationalization of indicators, the research team will develop technical specifications for indicators that are accepted or marked as “uncertain.” This will include the development of provisional numerators, denominators, exclusions, and data sources from which indicators can be measured in current primary care practices in England (Table S3 in Multimedia Appendix 1). For indicators proceeding to round 2 discussion, these technical specifications will be used to support the consensus meeting discussion.

### Round 2: Consensus Meeting

Controlled feedback, a classic feature of Delphi-type studies, will be provided to each panel member [44]. An individual feedback report will be sent 1 to 2 weeks prior to the round 2 consensus meeting. This will include participants’ round 1 ratings, anonymized comments from participants, and summary statistics (median and IQR) for each indicator by stakeholder group, presented using a color-coded system to indicate agreement status (Table S2 in Multimedia Appendix 1).

For patient participants, a lay summary will be coproduced with our community partners including participants’ own responses, the overall panel median without IQRs, and relevant free-text comments. Individual prior scores will not be disclosed to the wider group maintaining data anonymously.

The round 2 consensus meeting is a characteristic of RAM [33] and will consist of a fully online or hybrid consensus workshop, across 1 or 2 days, depending on participant availability [57]. Given the large initial set of candidate indicators and to reduce participant attrition, this meeting will review only those indicators marked as “uncertain” or demonstrating conceptual ambiguity or redundancy through free-text comments made in round 1.

At the start of the consensus meeting, all indicators marked as uncertain will be presented sequentially to the expert panel, each including a definition, proposed numerator, denominator, and data sources (Table S3 in Multimedia Appendix 1). Participants will independently rate each indicator in real time using an audience response system (eg, Teams voting polls), selecting one of three options: (1) accept indicator as an important measure of high quality of care for those with MLTCs, (2) indicator requires discussion before accepting or rejecting, (3) discard indicator as a not important measure of high quality of care. No discussion will take place during this silent voting process, ensuring balanced participation and preventing dominance of individual viewpoints, particularly between patients and health care providers.

Once all the panel votes on these indicators, predefined thresholds will be used to prioritize indicators discussion process within the meeting. Indicators receiving ≥60% agreement for accept will not be discussed further at this stage and will proceed directly to the final rerating round 3. Indicators receiving ≥40% “requires discussion” votes will be prioritized for structured discussion. Indicators receiving >20% “do not retain” votes will be discussed explicitly before any decision is made about removal. If indicators receive ≥60% reject votes, then the indicator is rejected without further discussion.

The panel will be asked to comment on clarity of definitions, wording refinements, and conceptual overlap with other indicators. HCP participants will be asked to consider the feasibility of operationalization, including the proposed numerator, denominator, and data sources. Patient and caregiver participants will be asked whether the proposed measure, including the numerator, reflects their lived experience and captures what matters to them in care.

Discussions will be audio-recorded and transcribed verbatim for analysis. All participants will have provided written informed consent before the meeting. Transcripts will be analyzed using content analysis, and all proposed modifications, decisions, and rationales will be documented to ensure transparency and reproducibility. Final consensus and prioritization will be determined in round 3.

### Round 3 Final Rerating and Consensus

Before round 3, participants will be sent via e-mail a feedback report summarizing the main changes made to the indicators, including the rationale for these decisions based on the meeting’s discussions.

During round 3, both stakeholder groups will rerate any indicators that were merged or revised as part of round 2 discussions. This will be conducted using the same web-based platform and 9-point Likert scale and consensus criteria as in round 1. Panelists will be asked to reconsider their ratings based on group discussion and feedback.

To address the operationalization of the indicator set, round 3 will also include specific questions on measurability and feasibility of all indicators reaching round 3 (Table 3). Patient participants will not be asked to rate these measurability and feasibility questions as advised by our CPs.

**Table 3.** Round 3 additional feasibility rating domains for expert health care professional participants.

Participant group and rating assessment criteria	Questions	Rating scale
Health care participants		
Interpretability and Measurability	Do you agree that the proposed numerator, denominator, exclusions, and data source are appropriate for measuring this indicator? Free-text comments.	Nine-point Likert scale, where 1 indicates “Strongly disagree,” 5 indicates “Partly agree,” and 9 indicates “Strongly agree.” Please suggest any changes to the numerator, denominator, exclusions, or data source.
Feasibility	How feasible would it be to measure this indicator using data currently available in routine primary care records or administrative systems? Free-text comments.	Nine-point Likert scale, where 1 indicates “Not feasible” (would require substantial EHR^a^ changes), 5 indicates “Partly feasible“ (some data available but some changes are needed”, and 9 indicates “Highly feasible” (can be measured using routinely recorded data with minimal additional burden). Please describe any data source, coding, EHR^a^ template, workflow, or implementation issue that may affect measurement.

a EHR: electronic health record.

Feasibility is being evaluated at this stage and separately from importance questions to distinguish indicators that are conceptually important from those that are likely to be immediately measurable or implementable in routine practice. Following round 3 results, the final set of indicators will be categorized according to their likely measurement readiness, following a red/amber/green system: those potentially measurable using existing routine primary care data (green), those requiring some changes to existing local templates and electronic health records (amber), and those requiring extensive changes and classed as difficult to change existing templates (red). This classification will guide subsequent feasibility testing and help assess the transferability of the indicator set across different English primary care settings.

At the conclusion of the RAM study, panel members will be invited to provide feedback on the process and the acceptability of the final indicator set [53].

### Ethical Considerations

This study was conducted in accordance with the principles of the Declaration of Helsinki. Ethical approval was obtained through the Imperial College Research Ethics Committee, acting as the institutional ethics review board, and administered by the Imperial College Research Governance and Integrity Team, with Head of Department approval on April 24, 2025 (Imperial College Research Ethics Committee reference 7594295). All participants will receive an information sheet and will be asked to sign a consent form. Survey data from Qualtrics will be collected in an anonymized format as per the platform’s inbuilt confidentiality settings. The consensus meeting recordings will be stored on secure, password-protected institutional servers and accessible only to authorized members of this research team. Recordings from consensus meetings will be transcribed, pseudonymized, checked for accuracy, and deleted after transcription and data analysis. Participants will be able to withdraw at any time, including between rounds. After their data are anonymized and synthesized into group-level findings, individual data may no longer be identifiable for removal. Between rounds, during participant feedback reports, only anonymized group-level findings will be reported to protect participant confidentiality.

## Results

This study received funding in June 2024. The scoping review component was finalized in January 2026 and published in May 2026. Qualitative interview data collection took place between May 2025 and July 2025, with data analysis concluded in December 2025. The associated manuscript reporting the qualitative findings is currently under review elsewhere.

As of July 2026, the RAM consensus process has a core group of 4 community advisory members, who will participate in all stages of this study. Additionally, 10 patient and caregiver participants and 12 HCPs have been consented. Data analysis and results from all 3 planned RAND/UCLA rounds are expected to be published in February 2027.

## Discussion

### Anticipated Principal Findings

This protocol describes a planned co-designed approach to develop primary care quality indicators for people living with MLTCs. This study will generate quality indicators that are grounded in published evidence and informed by patient and caregiver priorities. Through a 3-round RAM consensus process, indicators will be rated by both patients, caregivers, and expert health care professionals on importance, existing competing indicators, and feasibility on proposed numerators, denominators, and contemporary data sources embedded in existing primary care electronic health records. The final set of indicators will require further testing and validation in routine GP primary care settings in England.

### Comparison With Prior Work

Previous work has established important methodological foundations for developing quality indicators in primary care; however, their applicability to MLTC remains limited as they commonly focus on disease-specific care [58], older populations [26,59], or prescribing outputs [16].

A second limitation of previous indicator development is the variable and often limited involvement of patients and caregivers. A systematic review by Kötter et al [60] on the involvement of patients in quality of care indicator development found scarce involvement of patients in panels largely led by professional and technical professionals. Although patients are increasingly involved in consensus panels, they often remain numerically outnumbered by professional participants, with minimal reporting on ethnicity and socioeconomic characteristics [61]. Even when patients are actively involved, patient-prioritized aspects of care may be dismissed because they are perceived as too difficult to measure. For example, Pohontsch et al [62] found that aspects of care considered important to patients were often categorized as too “generic” in contrast to guideline-driven “hard outcome” metrics. Indeed, disease-specific indicators and clinical outcome measures remain important for monitoring population health and evidence-based care. However, especially in MLTCs, these measures need to coexist with indicators that capture microlevel interactions between patients and professionals, meso-level organizational arrangements such as access and coordination, and macro-level system conditions such as workforce capacity, digital infrastructure, and social support [63]. Over-reliance on incentivized disease-specific metrics risks reinforcing checklist-driven care and may contribute to changes in relational continuity and trust between HCPs and patients [64].

A further challenge concerns transferability across clinical, organizational, and national contexts. Although several quality indicators have been developed in the United States [22], Canada [23], and Germany [65], fewer are directly developed specifically for the UK care setting [16]. A systematic review of European studies on quality indicators for hospital readmission rates highlighted that indicators developed in one clinical, organizational, or national context cannot be assumed to transfer directly to another without attention to local pathways, data infrastructure, professional workflows, and patient priorities [66]. In the United Kingdom, the NICE multimorbidity quality standard [17] remains a useful starting point, but it is brief, with only four priority statements to function as a framework for assessing quality of care for people with MLTCs. When compared with broader patient-centered care quality indicator frameworks, its limitations are evident. The work of Santana et al [28] on person-centered care quality indicators identified a much wider set of 26 indicators across structure, process, outcome, and global assessment categories. The MULTIqual study by Schulze et al [59] further exposes this narrowness by validating a core set of 12 GP-reported and 7 patient-reported indicators in Germany’s primary care system, recognizing that quality in MLTCs must be assessed through professional report, patient experience, and documented clinical processes. Although not aiming to develop quality indicators, the European SELFIE conceptual framework for integrated care for multimorbidity also exposes NICE’s limitations. SELFIE, led by Leijten et al [67], organized care around the 6 adapted World Health Organization health-system components: service delivery, leadership and governance, workforce, financing, technologies and medical products, and information and research, across micro, meso, and macro levels. Therefore, where NICE offers a concise set of minimum standards, these frameworks suggest that quality in MLTCs should move beyond a small number of care-process statements and toward a layered model that includes patient-reported experience, professional assessment, organizational capability, information infrastructure, and system-level integration.

### Strengths and Limitations

One of this study’s strengths is the central role of patients and caregivers in indicator development. Rather than being invited only to comment on final outputs, patients and carers contribute to the conceptual framing of quality and to the design of the consensus process. Given the well-documented differences in priorities between patients and HCPs [13,68], particularly in MLTC care [69,70], the inclusion of patients and caregivers in consensus processes is essential to ensure that indicators reflect priorities that are meaningful to those receiving care, as well as those delivering it [41]. The study is also designed with future implementation in mind, including feasibility assessment of embedding proposed indicators in existing pathways and systems during rounds 2 and 3.

Nevertheless, this study also has limitations. First, there are no well-established quality indicators for MLTCs in primary care that are not specific to individual risk factors or conditions. The heterogeneity of the published literature developing or evaluating quality indicators in MLTC was scarce, with only 1 study developing well-explained methodologies and evaluating the validity of an indicator set [59,65]. Reliance on qualitative work means that substantial interpretive work is required to translate broad themes and subthemes into discrete, measurable candidate indicators. Consequently, several indicators may conceptually sit within more than one quality domain. Although this will be addressed through iterative discussion with the study team, core participants, and structured consensus methods, some overlap is likely to remain.

Finally, Delphi-type methods are inherently judgment-based, and particularly in MLTCs where consensus on its definition is still up for debate [71], different panels might reach somewhat different conclusions when presented with the same evidence, although some argue that this is not always the case [47]. To strengthen credibility and relevance, this study will include a broad group of knowledge users and apply a transparent rating process to combine empirical evidence with stakeholder expertise.

### Future Directions

The final indicator set deriving from this RAM study should be viewed as a consensus-based starting point that requires further validation, feasibility testing, and real-world evaluation rather than immediate implementation. While some indicators are likely to be more readily measurable, particularly those aligned with existing systems and quality statements, others will require new data capture or refinement once tested in practice. For example, indicators measuring medication reviews are already partially in place in many primary care electronic health records in the UK as part of NICE quality standards and therefore expected to be easier to achieve [17,72].

Future work will test the feasibility, transferability, and implementation of the final indicator set in routine GP settings in England. A Freedom of Information request to Integrated Care Boards in England has been initiated to understand how current quality indicators beyond nationally mandated QOF are currently captured, monitored, and used in practice, including data systems, governance, dashboards, and reporting processes that support local quality monitoring. Pooled findings from this request will identify which existing data sources and infrastructures could be used to operationalize the proposed indicators and where local variation may affect implementation across primary care services in England [73]. A freedom of information request rather than a survey or gray literature search was selected because locally developed dashboards and quality monitoring frameworks are not routinely described in the academic literature or publicly available. Surveys of policymakers and system leaders may be limited by low response rates [74].

Following this, qualitative work with local improvement leaders and electronic health record developers will explore how indicators can be operationalized, automated within routine data systems, and integrated into practice.

## Supplementary material

10.2196/99217 Multimedia Appendix 1 Quality of care indicator development additional information.

10.2196/99217 Checklist 1 GRIPP 2-Short Form.

## Abbreviations

CP: community partner
GP: general practice
GRIPP: Guidance for Reporting Involvement of Patients and the Public
HCP: health care professional
MLTC: multiple long-term condition
NICE: National Institute for Health and Care Excellence
QOF: Quality Outcomes Framework
RAM: RAND/UCLA Appropriateness Method
