# Supplementary material for: Codeveloping a Set of Quality of Care Indicators for People Living With Multiple Long-Term Conditions in Primary Care: Protocol for a Modified RAND/UCLA Study
Source: JMIR Res Protoc. 2026 Aug 7;15:e99217. doi: 10.2196/99217 (PMC13451058; doi:10.2196/99217)
Supplement: Supplementary file 1 [file resprot-v15-e99217-s001.docx]

**Table S1 – Example of triangulation table for indicator development deriving from scoping review findings and qualitative interviews**

| **Inductive Framework** | **Domain of quality** | **Scoping Review** | | **Qual Interview** | **Indicator Description (sub-domain)** | **Donabedian Component** |
| --- | --- | --- | --- | --- | --- | --- |
| **Patient and Carer Centredness** | **Information Access** | ✓ 10 studies | +/- 1 indicator (facilitating patient education and self-management) | ✓ | During clinical reviews, patients and caregivers have access and are provided with reliable sources of information which include leaflets, credible websites, condition-specific charities or programmes, peer-support groups, or digital tools (i.e. apps or patient portals) that are relevant to patient’s conditions or caregivers needs  ***Title: Access to patient education information sources*** | Structure |
|  |  | +/- partly present in 4 studies (Access to information about own health) | ✓ 1 indicator on anticipation of clinical needs (laboratory studies) | ✓ | Tests and investigation results requested by the GP surgery are shared in a format accessible to patients or caregivers and depending on their preferences. Communication may occur via digital tools (e.g., portals, apps, text messages) or direct discussion (e.g telephone or during reviews). Results, including those that are not clinically significant, are acknowledged and communicated. Results requiring urgent action should be communicated back to patients and caregivers within 24-48h hours depending on clinical urgency.  ***Title: Timely communication of test results*** | Structure / Processes |
|  | **Shared Decision-Making** | ✓ 13 studies | ✓ 2 indicators  ✓2 PREMs tool | ✓ | Decisions about treatments, medications, referrals, and tests are made collaboratively with patients. These should include information exchange on diagnosis, trade-offs between treatments and potential benefits, side-effects, prognosis and impact on quality of life. These discussions should also reflect alignment with patient’s preferences, priorities and needs  ***Title: Shared decision making with patients*** | Processes |
|  |  | ✓ 2 studies | ✓1 indicator | ✓ | Where an informal caregiver is involved in a patient’s care, they are appropriately included in decision-making discussions. Caregivers are provided with relevant information about the patient’s condition, treatment options, and potential impacts, and their concerns and capacity to support care are acknowledged, with the patient’s consent.  ***Title: Shared decision making with carers*** | Processes |

**Table S2 – Feedback report template to all participants after round 1 analysis**

|  | ***Quality Indicator Title*** | **Free-text comments** | **Patients (n = 7)** | | | | **HCPs (n=12)** | | | | | | | | | **Agreement?** |
| --- | --- | --- | --- | --- | --- | --- | --- | --- | --- | --- | --- | --- | --- | --- | --- | --- |
|  |  |  | **importance to pt** | | **Current practice** | | **importance quality** | | **Importance patients** | | **import clinical** | | **current practice** | | **Competing indicators** |  |
|  |  |  | **med** | **Iqr** | **med** | **iqr** | **med** | **iqr** | **med** | **iqr** | **med** | **iqr** | **med** | **iqr** | **(y/n)** |  |
| 1. | ***Regular and proactive monitoring*** | Anonymous comments can be incorporated as a part of the feedback here |  |  |  |  |  |  |  |  |  |  |  |  | If Yes, names provided here | Yes  No  Uncertain |

**Table S3 – Example of a quality indicator with operational definitions to be discussed at the consensus meeting**

| Indicator name | Definition | Technical definitions including  Numerator (achieved population)  Denominator (target / eligible population)  Data sources (where to obtain information) |
| --- | --- | --- |
| Allocated healthcare professional for medication review | There is a designated healthcare professional responsible for reviewing medication regime across all chronic conditions who is a qualified healthcare professional trained in physical assessment, independent prescribing and history taking (GP, pharmacist or advanced nurse practitioner). This person is the main contact in case of side-effects from medications. | **Denominator:** Total no. of patients with MLTC who have complex medication regimes., defined as: 1) Polypharmacy, specifically those on 10 or more medications; 2) Medicines commonly associated with medication errors; 3) severe frailty, particularly isolated or housebound or who have had recent hospital admissions and/or falls. Tools for audit and identification is provided on NHS SMR guidance.  **Numerator (structure):** No. of patients in denominator who have a clearly identified healthcare professional responsible for reviewing their entire medication regimen across all chronic conditions, with a named contact for advice or reporting side effects.  **Numerator (patient experience - outcome):** No. of surveyed patients in the denominator who report that they know who to contact about medication concerns, including side effects, interactions, or problems taking medicines.  **Data Sources:** Patient Experience survey, GP electronic health record system |
