# Supplementary material for: Codeveloping a Set of Quality of Care Indicators for People Living With Multiple Long-Term Conditions in Primary Care: Protocol for a Modified RAND/UCLA Study
Source: JMIR Res Protoc. 2026 Aug 7;15:e99217. doi: 10.2196/99217 (PMC13451058; doi:10.2196/99217)
Supplement: Supplementary file 2 [file resprot-v15-e99217-s002.docx]

**Supplementary file 2 – Community Partner Involvement (PPI) GRIPP 2-Short Form (SF)**

| **Section and topic** | **Item** |
| --- | --- |
| **1: Aim** Report the aim of the study | This study’s main aim is to co-develop with those affected by MLTC a set of quality-of-care indicators that can be pragmatically used within the NHS context to both measure and improve QoC delivery for this population. To meet this wider aim, we have also devised stepping goals for each step.  Goal 1: Understand what quality means in MLTC from patient and caregivers’ perspective.   - Goal 2: Co-develop a framework for conceptualising MLTC QoC from patient and caregiver perspective. - Goal 3: To prioritise a set of co-developed indicators according to their importance to patients and caregivers, relevance to clinical activity in primary care, alignment with patient-identified domains of quality, and feasibility for implementation within existing clinical pathways. |
| **2: Methods** Provide a clear description of the methods used for PPI in the study | **Study design and focus:**   - Community partners (CPs) helped shape the research focus and refine research questions, emphasising the need for indicators that reflect patient experience, address variation in care, and capture populations who may be excluded from routine services.   **Systematic review:**   - CPs reviewed the systematic review protocol and contributed to interpretation of thematic findings through structured discussions with the research team. Although we had given the option to participate in screening of included studies, our CPs found this to be too time consuming and not aligned with their expertise.   **Qualitative study:**   - CPs co-developed the interview topic guide, ensuring clarity and relevance for participants. They also supported recruitment through patient and community networks. - CPs suggested conducting individual interviews rather than focus groups, as these would allow deeper individual reflection and the sharing of more personal experiences of care that may be hindered in larger group settings.   **Between Review, Interviews and RAND/UCLA Appropriateness Method (RAM):**   - Indicator development: CPs contributed to the development and refinement of indicators initially developed through triangulation of review findings, qualitative data, and conceptual framing. We have met several times to ensure that definitions were congruent with qualitative interview findings and their own lived experiences with MLTC care.   **RAM study (planned involvement):**   - Survey development: CPs will support user-testing of the Round 1 survey, including development of lay descriptions, assessment of clarity, usability, and survey burden. - Between rounds: CPs will participate in a structured discussion to review survey findings, particularly areas of disagreement, and informed revisions to indicators. - Consensus meeting: CPs will equally participate in the consensus panel meeting/workshop to discuss and refine indicators that did not reach agreement during the survey.   **Dissemination**   - CPs contributed to scoping review and qualitative interviews manuscript preparation, poster presentations relating to this study, and particularly lay summaries. They are included as co-authors on all of these. |
| **3: Results** Outcomes—Report the results of PPI in the study, including both positive and negative outcomes | - **Conceptual and design impact:** During analysis of qualitative themes, CPs advised against mapping findings to pre-existing frameworks and supported an inductive analytic approach aligned with the study’s phenomenological orientation. - **Recruitment:** CPs networks facilitated recruitment of approximately half of qualitative interview participants. - **Data collection tools:** CP involvement improved the accessibility and clarity of study materials by reviewing and making extensive changes to participant-facing materials, such as patient information sheets, recruitment flyers, consent forms. They also co-developed interview guides into jargon-free language. - **Delphi process (planned): CPs** will contribute to identification and refinement of indicators between rounds, particularly those generating substantial participant feedback. - **Dissemination (planned):** CPs will contribute to manuscript development, particularly lay-facing sections, and continue to provide iterative feedback on drafts. They have reviewed this GRIPP form and made significant changes to discussion section below. |
| **4: Discussion** Outcomes—Comment on the extent to which PPI influenced the study overall. Describe positive and negative effects | Facilitators of CPs involvement:   - The patient partners have received training around in research methods and PPI. They are actively involved in a patient and public involvement group attached to Imperial College Patient Experience Research Centre (PERC). - The right processes were in place, as the patient partners were involved from the beginning of this project, allowing them to help shape it from the outset. Having the right context, with a collaborative research team, funding to finance their time, and a supportive attitude of their involvement from Imperial PERC, also positively assisted in the positive impact that PPI had on this study.   Challenges faced:   - Some indicators required substantial reinterpretation to ensure clarity and relevance from a lay perspective. This highlighted conflicts between clinical priorities and what matters most to patients. - The methods used to gain consensus on the upcoming RAM are quite strict. The RAM although systematic, does define the type of questions to be asked and the format of answers (i.e. 9 scale Likert – type). We anticipate that this inflexible methodology will create some challenges when seeking true co-production from our CPs. When we presented the planned RAM format, CPs have voiced frustration on the length of questionnaire involving questions to all anticipated 54 indicators. - Furthermore, the time for feedback between RAM rounds is ideally very short, so organising times where both researchers and patient partners could meet will be very difficult. |
| **5: Reflections** Comment critically on the study, reflecting on the things that went well and those that did not, so others can learn from this experience | - During the scoping review, although we had given the option to participate in screening of included studies, our CPs found this to be too time consuming and not aligned with their expertise. At this stage, we understood that we need to be attuned to CPs strengths, and allow individual members to participate only to the extent they feel comfortable and competent. - The sample recruited for the qualitative interviews was not fully representative of the wider MLTC population, being less ethnically diverse, younger, and generally more highly educated. This likely reflects the recruitment channels used and could be addressed through broader targeted recruitment strategies in future work. |
